# Validity and reliability of the Apple Health app on iPhone for measuring gait parameters in children, adults, and seniors

**DOI:** 10.1038/s41598-023-32550-3

**Published:** 2023-04-01

**Authors:** Christian Werner, Natalie Hezel, Fabienne Dongus, Jan Spielmann, Jan Mayer, Clemens Becker, Jürgen M. Bauer

**Affiliations:** 1 Geriatric Center, Agaplesion Bethanien Hospital Heidelberg, Heidelberg University Hospital, 69126 Heidelberg, Germany; 2 Institute of Sports and Sports Science, Heidelberg University, 69120 Heidelberg, Germany; 3 TSG ResearchLab, 74939 Zuzenhausen, Germany; 4 Unit of Digital Geriatric Medicine, Heidelberg University Hospital, 69115 Heidelberg, Germany

**Keywords:** Public health, Physical examination, Software, Biomarkers, Engineering

## Abstract

This study assessed the concurrent validity and test–retest-reliability of the Apple Health app on iPhone for measuring gait parameters in different age groups. Twenty-seven children, 28 adults and 28 seniors equipped with an iPhone completed a 6-min walk test (6MWT). Gait speed (GS), step length (SL), and double support time (DST) were extracted from the gait recordings of the Health app. Gait parameters were simultaneously collected with an inertial sensors system (APDM Mobility Lab) to assess concurrent validity. Test–retest reliability was assessed via a second iPhone-instrumented 6MWT 1 week later. Agreement of the Health App with the APDM Mobility Lab was good for GS in all age groups and for SL in adults/seniors, but poor to moderate for DST in all age groups and for SL in children. Consistency between repeated measurements was good to excellent for all gait parameters in adults/seniors, and moderate to good for GS and DST but poor for SL in children. The Health app on iPhone is reliable and valid for measuring GS and SL in adults and seniors. Careful interpretation is required when using the Health app in children and when measuring DST in general, as both have shown limited validity and/or reliability.

## Introduction

Walking is the most common form of human movement, and a safe and efficient gait is a prerequisite for independence across the life span. Poor gait has been shown to be a risk factor for falls, cognitive decline, disability, and mortality^1–4^. As such, gait is considered an important indicator of general health status^5,6^, highlighting the clinical relevance for regular gait assessments in healthcare setting. An individual’s gait is most frequently assessed by the speed of walking. However, gait is multidimensional and cannot be characterized by one parameter alone^7^. Quantifying other spatio-temporal gait characteristics gives a more detailed insight into the specific gait pattern and enables identification of gait disorders and underlying mechanisms. Additionally, step length and/or double support time, for instance, have been shown to predict adverse health outcomes such as falls, disability and mortality, independent of gait speed^8–10^.

Video motion capture systems, force platforms, and instrumented walkways are currently considered as the gold standards for quantitative gait analysis, but are expensive, resource intensive, and limited to stationary use in laboratory environments^11,12^. More affordable, easier to use, and less restrictive gait analysis methods are wearable sensor systems that rely on inertial measurement units (IMUs; accelerometer, gyroscope, and magnetometer) attached to different parts of an individual’s body^12^. They have proven to be valid and reliable alternatives to stationary laboratory systems and enable also out-of-laboratory gait analysis^13–18^. However, these systems still require specialized equipment (e.g., fixing material, host computer, access point) and in-person contact with trained personnel to operate (e.g., IMU attachment, test administration, data processing), and the test protocols focus on supervised and controlled conditions and cover only a limited period of time. Gait parameters obtained in such a way refer to how a person can optimally walk in a standardized environment (“gait capacity”)^19^, which has been shown to be only weakly related to how a person actually walks in daily-living environments ("gait performance")^20–23^. This weak relationship can be attributed to the subjects being more focused or over performing when there are no external distractions that require additional attention, or trying to walk as best they can when aware of being assessed (“Hawthorne effect”)^22,24^. Walking in daily life is more complex and influenced by various environmental factors not present under controlled conditions. Measurements over a short period of time (“snapshot observations”) also cannot monitor acute changes when they occur or distinguish between acute changes and slower changes over time.

Recent advances in sensor technology have led to wearable sensors that now allow for more unobtrusive and continuous remote gait monitoring over longer periods of time while walking freely and unsupervised in daily life^24–26^. A stand-alone IMU is usually placed in the lumbar region close to the subject's center of mass with a special elastic belt or adhesive skin tape and worn continuously over several days^1,20,27,28^. After the measurement period, the sensor is removed and the raw data is processed and analyzed with offline software tools. While these IMUs enable daily-life gait monitoring, they are not yet easily accessible, depend on the need of specialized equipment, willingness and acceptance to wear the sensor and technical knowledge for data processing, and do not provide subjects with immediate feedback on their gait performance.

Smartphones have become an almost integral part of human life. The number of global smartphone users is estimated to be about 6.6 billion by 2022, suggesting that more than 80% of the world's population owns a smartphone^29^. Nowadays, these ubiquitous mobile electronic devices are usually embedded with IMUs that can also be used for unobtrusive and continuous daily-life gait monitoring. Given the ubiquity, easy accessibility and high acceptability of smartphones, applications (apps) that use these built-in IMUs for gait monitoring combined with easy-to-use interfaces, automatic data processing and analysis, and real-time reporting on gait performance may overcome the limitations of stand-alone IMUs. There is emerging evidence for the validity and/or reliability of smartphone apps to quantify spatio-temporal gait parameters in children and adolescents^30^, young and/or older adults^31–38^, and patients with (neuro-)muscular pathologies^39–42^. Apart from a few exceptions (e.g., Apple Health, OneStep, Gait Analyzer), most of these apps still focus on standardized measurements of gait capacity by providing multimedia instructions for self-administered test protocols^34–36,39,40^ and depend a standardized smartphone placement (e.g., sacroiliac belt)^35,36,39,42^.

The freely accessible Apple Health app (a.k.a. HealthKit) on iPhone offers a passive, unobtrusive and fully automated method for measuring the user’s daily-life gait performance, while wearing the iPhone in the pocket and without conscious measurement initiation. Spatio-temporal gait parameters are provided in real time on a user-friendly interface and are visualized in interactive charts to review progress over time. Concurrent validity of the Health app for measuring gait speed, step length, and double support time has recently been documented with a gold-standard instrumented walkway system in seniors^38^. Minimal detectable changes (MDCs) for inter-device reliability of these gait parameters have also been reported in this age group. To our knowledge, however, there is no evidence on the psychometric properties of the Health app in younger populations such as children, adolescents, and adults in young or middle age, which are more likely to own an iPhone than seniors^43^. In addition, and independent of the age group, the test–retest reliability of the Health app is still unknown. Since the Health app aims at continuous monitoring of daily-life gait performance and changes, knowledge about the stability of its measures over time in individuals with unchanged gait is, however, essential to determine if a real change in gait has occurred.

Therefore, the aim of this study was to assess the concurrent validity with a multi-IMU-based gait analysis system and the test–retest reliability of the Health app on iPhone for measuring gait speed, step length, and double support time in children, adults, and seniors.

## Methods

### Study design

This observational study was conducted from March 2022 to August 2022. A cross-sectional design was used to assess the concurrent validity of the Health app. Test–retest reliability was assessed using a prospective design with two testing sessions 1 week apart. The study was conducted according to the guidelines of the Declaration of Helsinki, and approved by the Ethics Committee of the Medical Faculty of Heidelberg (S-042/2022, 1 Jan 2022). Written informed consent was obtained from all participants (and legal guardians of participants under 18 years) prior to study inclusion. The study was prospectively registered at the German Clinical Trials Register (DRKS00028074).

### Study population

Three groups of participants were recruited: (1) children between 12 and 17 years from the youth teams of a professional soccer club (TSG Hoffenheim e.V.), (2) adults between 18 and 64 years from the children’s parents, the acquaintances of the research team, and the staff of a German geriatric hospital, and (3) seniors ≥ 65 years from healthy populations of previous studies conducted at the study center. Inclusion criteria were age ≥ 12 years, adequate German language skills, ability to perform the physical tasks and to understand study instructions, and written informed consent (from legal guardians). Exclusion criteria were severe musculoskeletal, cardiovascular, neurologic, sensory, cognitive, or psychiatric disorders and acute illness or injury.

The sample size was estimated to be *n* ≥ 27 in each age group, based on a prior power analysis for the agreement between methods (Health app vs. APDM Mobility Lab) and repeated measurements (test vs. retest), with an expected ICC of 0.90 and an acceptable ICC of 0.70 for two measurements (k = 2, Health app vs. APDM Mobility Lab), a statistical power (1 − β) of 0.80, and a significance level (α) of 0.05^44^, with a possible dropout rate of 15%.

### Descriptive measures

Demographic and clinical characteristics including age, gender and chronic diseases (yes vs. no) were obtained by standardized interview. Weight status was evaluated using the body mass index (BMI) and categorized into underweight, normal weight, and overweight^45^. Cognitive status was assessed with the Short Orientation-Memory-Concentration Test^46^. Self-reported health status was determined by the interview form of the EuroQol 5-Dimensions 3-Levels questionnaire (EQ-5D-3L) and the EQ visual analogue scale (EQ VAS)^47,48^. Physical function was measured using handgrip strength (JAMAR® PLUS + Dynamometer, Performance Health Supply Inc., Cedarburg, WI, USA)^49^, which were categorized into low, normal, and high according to European (children)^50^ or German (adults and seniors)^51^ normative values. Physical activity (PA) was assessed using the International Physical Activity Questionnaire-Short Form (IPAQ-SF)^52^. Following the IPAQ-SF scoring protocol, PA levels were categorized into low, moderate, and high^53^. All questionnaires and test procedures were conducted under standardized conditions by a research assistant who previously received extensive training in their administration.

### Six-minute walk test

Gait parameters were captured via the Health app during a 6-min walk test (6MWT), completed by participants with an iPhone (model: SE 64 GB, iOS software version: 15.4) in the right front pocket of their pants, with the display facing towards the participant. Participants were instructed to walk continuously at their usual, comfortable pace for 6 min along a flat and straight 20-m walkway with cones placed at each end to indicate turnaround points. The turning direction was standardized to walk around the cones counterclockwise. For each participant, the test administrator entered the personal information (date of birth, gender, height, weight) into the Health app before putting the iPhone in the participant’s pocket, made a note of the exact time for the start of the 6MWT, and measured the total walking distance via the lap numbers and a tape measure. Before the start signal and after the end signal, participants were asked to stand still for 10 s to facilitate the identification of the walking bouts for their 6MWT in the subsequent data extraction and processing in the Health app.

All children and some of the adult participants (parents of the children, acquaintances of the research team) completed the 6MWT outdoors on a firm and flat ground at the training site of the professional soccer club. Adults recruited from the hospital staff and all seniors completed the 6MWT indoors on a long and wide hallway within the geriatric hospital.

### Apple Health app

From the 8th generation of the iPhone with its built-in IMUs and the mobile operating system iOS 14, this pre-installed health and fitness app from Apple Inc. (Cupertino, CA, USA) passively and unobtrusively record daily-life gait performance of the iPhone user. When the iPhone is worn at waist level (e.g., in the pant pocket) and the user walks at a steady pace on a flat ground, the Health app automatically identifies a walking bout and provides mean values for the gait speed (m/s), step length (cm), and double support time (% of the gait cycle with two feet on the ground) of the walking bout. These gait parameters are derived from a biomechanical model of walking that depends on the leg length estimated from the user's height. Thus, to obtain the most accurate parameter estimates, the user needs to enter the height into the Health app^38^. The Health app does not support manual data processing prior to automatic analysis (e.g. for excluding turning movements), nor does it allow access to individual gait cycles, but automatically provides only the mean values of gait parameters for the identified walking bouts. Detailed information about the biomechanical model and algorithms used to estimate these gait parameters have not yet been published by Apple Inc. The app has an age rating of ≥ 12 years^54^.

For each 6MWT of a participant, the gait parameters automatically estimated by the Health app were extracted as follows: Data were exported in XML format to a local PC for further data processing^55^. The XML file was then imported into Microsoft Excel (version 16.64; Microsoft Corp, Redmond, WA, USA). Based on the 6MWT start time noted by the test administrator and the time stamps for the start and end of gait recordings provided in the XLSX file, walking bouts for each participant's specific 6MWT were identified and the corresponding mean values of gait speed, step length, and double support time were extracted.

### Procedure for testing the concurrent validity and test–retest reliability

To assess the concurrent validity of the Health app, gait parameters was additionally measured during the 6MWT using the APDM Mobility Lab (APDM Inc., Portland, OR, USA) as a reference standard, which has been successfully validated in children, adults, and seniors^13–15^. Simultaneously to the iPhone worn in the pant pocket, three synchronized Opal IMUs (size = 55 × 40.2 × 12.5 mm, weight < 25 g) were attached with straps bilaterally on both feet and the fifth lumbar vertebra. The Opal IMUs include two 3-axial accelerometers (range: ± 16 g and ± 200 g, resolution: 14 and 17.5 bits), gyroscope (range: ± 2000°/s, resolution: 12 bits) and magnetometer (range: ± 8 Gauss, resolution 12 bits) and record at a sampling frequency of 128 Hz. The APDM Mobility Lab uses radio-frequency communication for wireless data transmission and synchronization of the multiple Opal IMUs through an access point connected to a host computer. A test protocol for the 6MWT was designed within the APDM Mobility Lab software (V2.0.0.201903301644), where after pressing start by the test administrator the time automatically runs down to an auditory stop signal. Start and stop signals were loudly forwarded to the participants by the test administrator. APDM Mobility Lab software was used to automatically analyze the recorded data and extract mean values of gait speed (m/s), step length (cm), and total double support time (%). Turning steps are not included in the gait parameter extraction by the software, but only straight ahead walking.

To assess the test–retest reliability of the Health app, the 6MWT instrumented with the iPhone was repeated 1 week (6.9 ± 0.5 days) after the first test session. This retest was performed under the same conditions as the first one (i.e. same test environment, test administrator and iPhone placement).

### Statistical analysis

Descriptive data were presented as frequency and percentage, median and interquartile range (IQR), or mean and standard deviation (SD). The level of agreement between methods (Health app vs. APDM Mobility Lab) and repeated measurements (test vs. retest) for capturing gait speed, step time, and double support time were assessed by calculating systematic differences (bias) with 95% confidence intervals (CI), 95% limits of agreement (LOA = mean_bias_ ± 1.96 × SD_bias_), and intraclass correlation coefficients (ICC_2,1_, absolute agreement) with 95% CI. ICCs were interpreted as poor (< 0.50), moderate (0.50 < 0.75), good (0.75 < 0.90), or excellent (≥ 0.90)^56^. Bland–Altman plots were also constructed to visualize the level of agreement^57^. Percentage errors (PE) of the Health app compared to the APDM Mobility Lab were calculated by dividing the 1.96 × SD_bias_ by the mean for both methods and were considered to be clinically acceptable if < 30%^58^. Standard errors of measurement (SEM) were calculated by the square root of the mean square error terms from repeated-measures analyses of variance between the test–retest measurements^59^. MDCs at the 95% CI were calculated as SEM × 1.96 × √2. SEM% and MDC_95_% were also calculated as a percentage of the mean of test–retest measurements. SEM% were considered as low (≤ 10%) or high (> 10%), and MDC% as acceptable if < 30%^60,61^. Statistical analyses were performed using IBM SPSS Statistics, Version 27.0 (IBM Corp., Armonk, NY, USA).

## Results

### Participant characteristics

The total sample included 83 participants: 27 children (14.0 ± 1.5 years), 28 adults (31.3 ± 11.3 years), and 28 seniors (75.6 ± 5.7 years). Only two seniors reported having chronic diseases. More than two thirds in each age group (68–85%) could be classified as normal-weighted. Self-reported health status was good to excellent, with mean EQ-5D-5L indexes of ≥ 0.93 points and mean EQ-VAS scores of ≥ 72.1 points in all three age groups. More than 90% of participants (75 out of 83) showed normal to high handgrip strength. PA levels indicate an overall physically active sample. Further participant characteristics are shown in Table 1.

**Table 1:** Participant characteristics.

Characteristics	Children (*n* = 27)	Adults (*n* = 28)	Seniors (*n* = 28)
Age (years)	14.0 ± 1.5	31.3 ± 11.3	75.6 ± 5.7
Sex (female)	17 (63.0)	17 (60.7)	22 (78.6)
Chronic diseases (yes)	0 (0.0)	0 (0.0)	2 (7.1)
BMI (kg/m^2^)	20.2 ± 5.3	23.7 ± 2.9	23.5 ± 2.7
Underweight	0 (0.0)	0 (0.0)	1 (3.6)
Normal weight	23 (85.2)	22 (78.6)	19 (67.9)
Overweight	4 (14.8)	6 (21.4)	8 (28.6)
SOMCT (pt.)	2.0 [4.0–6.0]	0.0 [0.0–2.0]	2.5 [0.0–4.0]
Handgrip strength (kg)	31.4 ± 6.1	42.2 ± 10.1	27.4 ± 9.1
Low	0 (0.0)	1 (3.6)	7 (25.0)
Normal	15 (55.6)	22 (78.6)	19 (67.9)
High	12 (44.4)	5 (17.9)	2 (7.1)
EQ-5D-3L (pt.)	1.00 ± 0.1	0.99 ± 0.03	0.93 ± 0.10
EQ VAS (pt.)	88.7 ± 12.3	86.9 ± 8.3	72.1 ± 5.2
IPAQ-SF (MET-min/week)	4880 [4096–5514]	5529 [3287–7230]	6031 [3309–9171]
Low PAL	2 (7.4)	0 (0.0)	1 (3.6)
Moderate PAL	2 (7.4)	4 (4.3)	6 (21.4)
High PAL	23 (85.2)	24 (85.7)	21 (75.0)

Data given as *n* (%), median [interquartile range], or mean ± standard deviation.

*BMI* body mass index, *SOMCT* Short Orientation-Memory-Concentration Test, *EQ-5D-3L* EuroQol 5-Dimensions 3-Levels questionnaire, *EQ VAS* EuroQol visual analogue scale, *IPAQ-SF* International Physical Activity Questionnaire-Short Form, *PAL* physical activity level.

### Concurrent validity

The Health app provided no gait data for four participants (4.8%, children: *n* = 3, adults: *n* = 1) and no data on double support time for another 13 participants (15.7%, children: *n* = 6, adults: *n* = 3, seniors: *n* = 4). No missing data were observed for the gait data captured with the APDM Mobility Lab.

**Figure 1:**
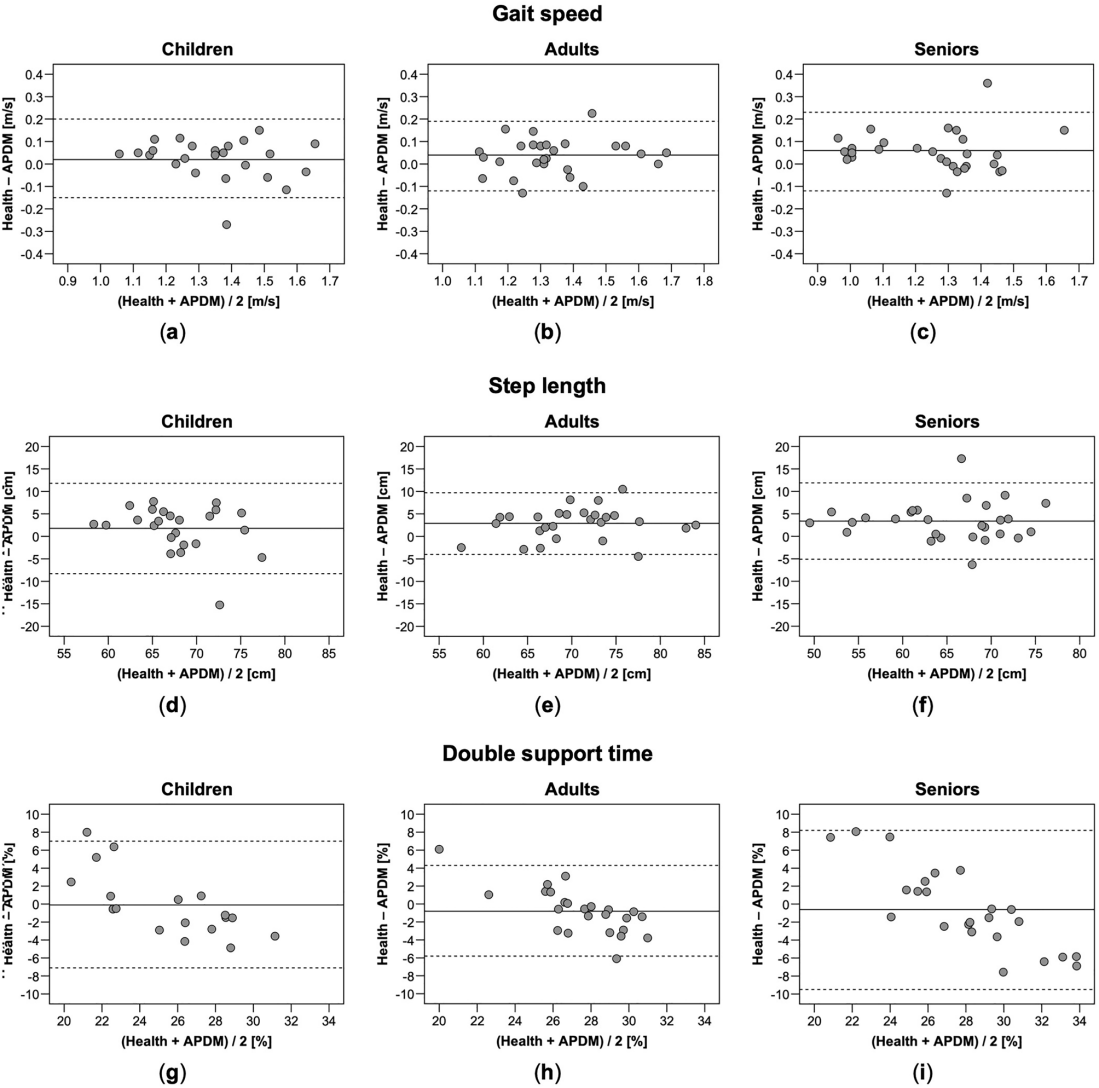
Bland–Altman plots for gait speed (**a**–**c**), step length (**d**–**f**) and double support time (**g**–**i**) measured with the Health app and the APDM Mobility Lab in children, adults, and seniors. Solid lines indicate mean between-method differences (bias) and dashed lines indicate upper and lower 95% limits of agreement.

Level of absolute agreement between the Health app and the APDM Mobility Lab was highest for gait speed, with clinically acceptable PEs (11.6–14.1%) and good ICCs ranging from 0.85 to 0.86 in all three age groups (Table 2). Agreement was lower for step length, with clinically acceptable PEs (9.8–14.8%) in all three age groups and good ICCs in adults (0.78) and seniors (0.76), but only a moderate ICC (0.53) in children. The lowest level of agreement was observed for double support time: PEs were clinically acceptable in children (27.7%) and adults (18.4%) but not in seniors (31.6%); ICCs ranged from poor in seniors (0.42) to moderate in children (0.54) and adults (0.58). Bland–Altman plots for agreement between the Health app and the APDM Mobility Lab revealed no systematic pattern of bias with increasing or decreasing values (Fig. 1a–i).

**Table 2:** Concurrent validity of the Health app with the APDM Mobility Lab.

Parameter	Apple Health app	APDM Mobility Lab	Bias (95% CI)	95% LOA	PE	ICC_2,1_ (95% CI)
Gait speed (m/s)						
Children (*n* = 24)	1.36 ± 0.16	1.34 ± 0.18	0.02 (− 0.01 to 0.06)	− 0.15 to 0.20	13.0	0.86 (0.70–0.94)
Adults (*n* = 27)	1.36 ± 0.17	1.32 ± 0.16	0.04 (0.00 to 0.07)	− 0.12 to 0.19	11.6	0.86 (0.70–0.94)
Seniors (*n* = 29)	1.28 ± 0.19	1.23 ± 0.19	0.06 (0.02 to 0.09)	− 0.12 to 0.23	14.1	0.85 (0.61–0.94)
Step length (cm)						
Children (*n* = 24)	68.9 ± 4.7	67.1 ± 6.0	1.8 (− 0.4 to 4.0)	− 8.3 to 11.8	14.8	0.53 (0.18–0.76)
Adults (*n* = 27)	71.8 ± 6.8	69.0 ± 6.2	2.9 (1.5 to 4.3)	− 4.0 to 9.7	9.8	0.78 (0.34–0.92)
Seniors (*n* = 29)	66.6 ± 7.4	63.2 ± 7.5	3.4 (1.7 to 5.1)	–5.1 to11.9	13.1	0.76 (0.32–0.90)
Double support time (%)						
Children (*n* = 18)	25.4 ± 2.3	25.5 ± 4.7	− 0.1 (− 1.9 to 1.7)	− 7.1 to 7.0	27.7	0.54 (0.09–0.80)
Adults (*n* = 24)	27.1 ± 1.8	27.9 ± 3.6	− 0.8 (− 1.8 to 0.3)	− 5.8 to 4.3	18.4	0.58 (0.25–0.79)
Seniors (*n* = 24)	27.6 ± 2.0	28.3 ± 5.5	− 0.6 (− 2.5 to 1.3)	− 9.5 to 8.2	31.6	0.42 (0.02–0.70)

Descriptive data given as mean ± standard deviation.

*CI* confidence interval, *LOA* limits of agreement, *PE* percentage error, *ICC* intraclass correlation coefficient.

### Test–retest reliability

Seven participants (8.4%, children: *n* = 6, seniors: *n* = 1) could not participate in the repeated 6MWT 1 week after the first test due to COVID-19, other acute diseases, or injuries. Out of the 76 retest measurements performed, the Health app provided no gait data for 16 participants (21.1%; children: *n* = 6, adults: *n* = 7, seniors: *n* = 3) and no data on double support time for another 18 participants (44.7%; children: *n* = 5, adults: *n* = 8, seniors: *n* = 3). Considering the missing data of four participants at the first test, test–retest reliability were analyzed for gait speed and step length in 56 participants (67.5%, children: *n* = 12, adults: *n* = 20, seniors: *n* = 24) and for double support time in 38 participants (45.8%, children: *n* = 7, adults: *n* = 10, seniors: *n* = 21).

**Figure 2:**
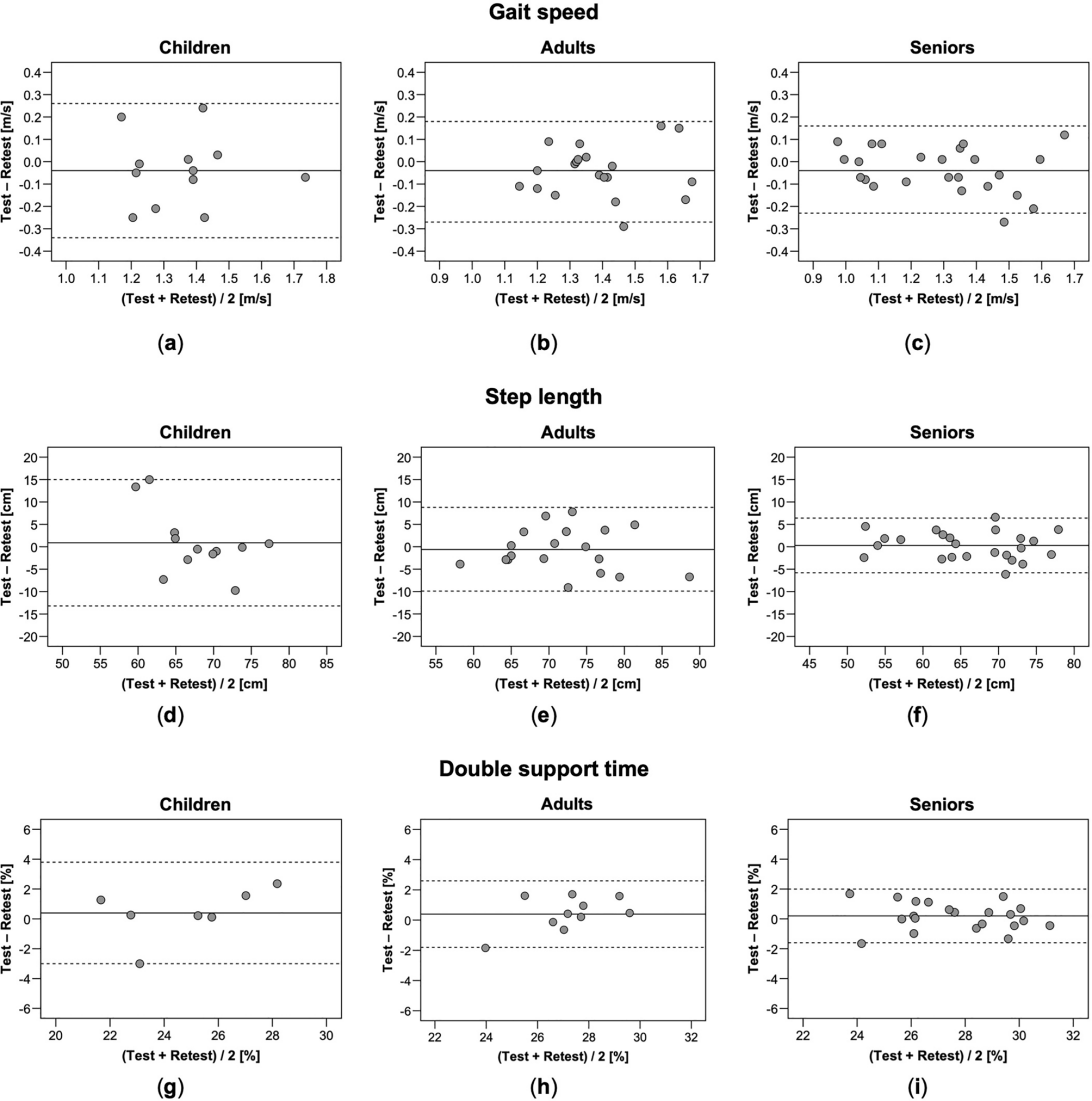
Bland–Altman plots for repeated measurements of gait speed (**a**–**c**), step length (**d**–**f**) and double support time (**g**–**i**) measured with the Health app in children, adults, and seniors. Solid lines indicate mean between-method differences (bias) and dashed lines indicate upper and lower 95% limits of agreement.

Consistently good to excellent ICCs between the repeated measurements were obtained for gait speed, step length, and double support time in adults (0.75–0.80) and seniors (0.88–0.93) (Table 3). In children, ICCs were moderate to good for gait speed (0.61) and double support time (0.79) but only poor for step length (0.39). Low SEM% (2.4–8.1%) and acceptable MDC_95_% (6.5–22.4%) were found for all gait parameters in all age groups, being lowest in seniors. MDC_95_ ranged from 0.20 to 0.30 m/s for gait speed, from 6.1 to 14.1 cm for step length, and from 1.8 to 3.4% for double support time. In general, test–retest differences (bias, 95% LOA), SEMs, and MDCs were larger in children than adults and seniors. Bland–Altman plots did not indicate systematic patterns of bias for the agreement between the repeated measurements (Fig. 2a–i).

**Table 3:** Test–retest reliability of the Health app.

Parameter	Test	Retest	Bias (95% CI)	95% LOA	ICC_2,1_ (95% CI)	SEM (SEM%)	MDC_95_ (MDC_95_%)
Gait speed (m/s)							
Children (*n* = 12)	1.34 ± 0.15	1.38 ± 0.17	− 0.04 (− 0.14 to 0.06)	− 0.34 to 0.26	0.61 (0.10–0.87)	0.11 (8.1)	0.30 (22.4)
Adults (*n* = 20)	1.37 ± 0.17	1.41 ± 0.16	− 0.04 (− 0.10 to 0.01)	− 0.27 to 0.18	0.75 (0.47–0.89)	0.08 (5.8)	0.22 (16.0)
Seniors (*n* = 24)	1.27 ± 0.20	1.31 ± 0.23	− 0.04 (− 0.08 to 0.01)	− 0.23 to 0.16	0.88 (0.74–0.95)	0.07 (5.5)	0.20 (15.2)
Step length (cm)							
Children (*n* = 12)	68.2 ± 4.5	67.3 ± 7.9	0.9 (− 3.7 to 5.5)	− 13.2 to 15.0	0.39 (0.25–0.78)	5.1 (7.5)	14.1 (20.8)
Adults (*n* = 20)	71.7 ± 7.7	72.2 ± 7.6	− 0.6 (− 2.8 to 1.7)	− 9.9 to 8.8	0.80 (0.57–0.92)	3.4 (4.7)	9.4 (13.0)
Seniors (*n* = 24)	66.2 ± 7.7	65.9 ± 8.1	0.3 (− 1.2 to 2.0)	− 5.8 to 6.4	0.93 (0.84–0.97)	2.2 (3.3)	6.1 (9.2)
Double support time (%)							
Children (*n* = 7)	25.0 ± 2.9	24.6 ± 2.1	0.4 (− 1.2 to 2.0)	− 3.0 to 3.8	0.79 (0.20–0.96)	1.2 (4.9)	3.4 (13.5)
Adults (*n* = 10)	27.4 ± 2.0	27.0 ± 1.4	0.4 (− 0.4 to 1.2)	− 1.8 to 2.6	0.78 (0.37–0.94)	0.8 (2.9)	2.2 (8.1)
Seniors (*n* = 21)	27.8 ± 2.1	27.6 ± 2.2	0.2 (− 0.2 to 0.6)	− 1.6 to 2.0	0.91 (0.80–0.96)	0.7 (2.4)	1.8 (6.5)

Descriptive data given as mean ± standard deviation.

*CI* confidence interval, *LOA* limits of agreement, *ICC* intraclass correlation coefficient, *SEM* standard error of measurement, *MDC* minimal detectable change.

## Discussion

The study assessed the concurrent validity of the Health app on iPhone with the APDM Mobility Lab, and its test–retest reliability over 1 week for measuring spatio-temporal gait parameters in children, adults, and seniors. To our knowledge, this is the first to provide evidence on the psychometric properties of the Health app’s gait measurements in different age groups.

Gait parameters were captured during a 6MWT that participants completed at usual gait speed. Considering the actual use case of the Health app for measuring daily-life gait performance, the 6MWT was selected as it better reflects real-life walking behavior than other clinically established walking tests^62,63^, and usual pace was prescribed as it is stronger associated with daily-life gait than fast pace^64^. In addition, a 6MWT has also been used in the previous study by Apple Inc.^38^ to test the concurrent validity of the Health app in seniors.

Of the total 159 measurements performed across the test and retest sessions, the Health app provided no gait data for 20 participants (12.6%) and no data on double support time for 51 participants (32.1%), with most missing data observed in children. A potential explanation for these findings might be the non-standardization of the participants’ pants during the 6MWT. Looser pants with larger pockets may have led to more artefactual smartphone movements and/or greater deviations from the participant’s center of mass, providing a poorer signal for detecting gait events and data processing. Indeed, such loose coupling of the iPhone has been seen more frequently in children. Missing data for the double support time could also be related to this procedure for wearing the iPhone. While the measurement of the other gait parameters is based solely on the detection of heel-strikes, that of double support time is based on the detection of both heel-strikes and toe-offs. Issues with the detection of one of these events may result in the double support time not being detected and calculated. Given that the toe-off event also occurs during a smoother foot movement and IMUs usually show greater errors in detecting this event^65–67^, double support time might also be more prone to non-detection than the other parameters when the iPhone is worn under non-optimal conditions. Overall, these findings support the recommendations of Apple Inc.^38^ that a good signal is required for the availability of gait measurements from the Health app by tightly coupling the iPhone to the user's center of mass.

Concurrent validity of the Health app was assessed against a well-established, multi-IMU-based system for gait analysis (APDM Mobility Lab)^13–15^. Level of agreement between the two methods was good for gait speed in all age groups (ICC ≥ 0.85). Lower agreement was observed for step length, which was still good in adults and seniors (ICC > 0.76), but moderate in children (ICC = 0.53). For double support time, the agreement was lowest and only poor to moderate (ICC = 0.42–0.58). These findings correspond to those obtained by Apple Inc.^38^ for the validity of the Health app against an instrumented walkway in seniors, with agreement levels that were good to excellent for gait speed (ICC = 0.92) and step length (ICC = 0.84), but moderate for double support time (ICC = 0.53).

Previous studies on the validity of another smartphone app allowing for unobtrusive daily-life gait monitoring (OneStep) also revealed similar findings in healthy adults^32^ and patients with musculoskeletal pathologies^41^. Agreement of the OneStep app with different reference standards (APDM Mobility Lab, Zeno walkway) was higher for gait speed (ICC = 0.94, Pearson correlation coefficient [*r*] = 0.89–0.91) and step length (ICC = 0.80, *r* = 0.65–0.84) than for double limb support (ICC = 0.52, *r* = 0.61–0.62). The lower validity for measuring double support time has also been reported for stand-alone IMUs in normal^68^ and pathological gait^69,70^. As previously discussed, the difficulty of accurately detecting both heel strike and toe-off for estimating this gait parameter has often been mentioned in this context as a potential reason for greater measurement errors with this gait parameter^65–67^, which may also be the case for the Health app. Furthermore, it is not completely clear if this finding is a limitation of the Health app or possibly of the reference standard, as the APDM Mobility has also demonstrated limited validity for measuring double support time^15^. However, as similar agreement levels have been reported for this gait parameter between the Health app (ICC = 0.53) and an instrumented walkway^38^, we do not assume that this finding relates to the limitation of the APDM Mobility Lab.

Apple Inc.^38^ reported slightly higher agreement levels of the Health app with the reference standard (ICC = 0.53–0.92) compared with the present study (ICC = 0.42–0.86). This might be related to the fact that due to not having access to the individual gait cycles in the Health app, we were not able to precisely time align them with the reference standard before data analysis, as was done by Apple Inc^38^. Thus, the mean values provided by the automatic analyses of the Health app and the APDM Mobility Lab for the gait parameters may not have been based on exactly the same gait cycles within the 6MWT, which could have affected the agreement level between the two systems.

Validity statistics for measuring gait speed and/or step length were quite similar or only slightly lower than previously observed for the Health app in seniors (ICC = 0.84–0.92)^38^, and for the OneStep (ICC = 0.80–0.96)^32,33^ and Gait Analyzer apps in adults (mean bias: gait speed = − 0.09 to 0.05 m/s, step length = − 4.0 to 2.3 cm)^37^. The lower validity might be due to the fact that these studies used fixing material for smartphone positioning (pocket holding, waist bag, hip clip)^32,33,37,38^, which has been shown to provide higher validity for smartphone-based gait analysis than when wearing it in the pant pocket^71^, as was done in the current study.

Compared with the concurrent validity reported for stand-alone IMUs, the Health app showed similar levels of agreement with the reference standard for measuring gait speed and step length in adults (ICC = 0.83–0.92, PE = 12.3–15.1%)^68,72^. In seniors, similar (ICC = 0.79–0.95, PE = 12.5–14.7%)^72,73^ but in some cases also substantial higher agreement levels (ICC = 0.99, PE = 2.1–2.3%^74^; mean bias: gait speed = 0.02 ± 0.02 m/s, step length = –0.59 ± 0.87 cm^75^) have been reported for these gait measurements via such IMUs.

The lower validity of the Health app obtained for children’s step length might be explained by their looser pants with larger pockets which may have been associated with more artefactual smartphone movements and thus greater measurement bias. Another potential explanation might be that the biomechanical model of walking from which the gait parameters are derived by the Health app may be based on the anthropometrics of adults rather than children and adolescents.

Test–retest reliability of the Health app was consistently good to excellent for all gait parameters in adults (ICC = 0.75–0.80) and seniors (ICC = 0.88–0.93). These findings correspond to those reported for the OneStep and Gait Analyzer apps, which also demonstrated good to excellent test–retest reliability for measuring gait speed (ICC = 0.77–0.98), step length (ICC = 0.80–0.97), and/or double support time (ICC = 0.90–0.98) in adults^31,33,37^. In contrast, reliability of the Health app was poor to moderate for measuring gait speed and step length in children (ICC = 0.39–0.61), which was lower than that of the Gait Analyzer app in children and adolescents (ICC = 0.87–0.94)^30^. A potential explanation for the lower reliability in children compared to the adults and seniors in the current study and to the Gait Analyzer app might be the fact that our children performed the 6MWT outdoors. These test conditions may have been more variable across the repeated measurements due to partially uncontrollable contextual and environmental factors (e.g. weather conditions, people passing by) than those indoors that prevailed for most adults and all seniors, as well as for testing the test–retest reliability of the Gait Analyzer app^30^. In general, however, our reliability results in children also have to be interpreted with caution due to the small sample size.

SEMs were calculated to obtain within-subject variability that typically occurs due to random measurement error. To our knowledge, such information on SEM has not yet been reported for the Health App or any other smartphone app that enable daily-life gait monitoring. For smartphone apps focusing on standardized gait capacity measurements slightly lower SEMs and SEM% were reported in healthy adults (SEM%: gait speed = 2.0–3.8%, step length = 2.5–4.0%)^36,42^ and patients with neurological diseases (SEM%: gait speed = 4.8%, step length = 2.5%^39^; SEM: gait speed = 0.01–0.02 m/s^42^). SEM% of the Health app were, however, lower than 10% for all gait parameters and in all age groups, which has often been considered to be a small amount of random measurement error^42,60,61^.

Based on the SEM, MDC_95_ and MDC_95_% values were calculated for each gait parameters captured with the Health app. These values provide the opportunity to determine if a real change has occurred that exceeds the measurement error or within-subject variability; thus, making them highly relevant for detecting changes over time or evaluating treatment effects. MDC_95_% for gait speed, step length and double support time were acceptable in all age groups (6.5–22.4%), suggesting that the Health app might be sensitive to detect changes in these gait parameters. MDC_95_ in seniors were similar to those previously reported by Apple Inc^38^. for gait speed (0.08–0.23 m/s), step length (4–12 cm), and double support time (2.1–4.5%). We extend these findings for the Health app in adults and children, who showed slightly larger MDC_95_ for gait speed (0.22–0.30 m/s) and step length (9.4–14.1 cm) than seniors (0.20 m/s, 6.1 cm). MDC_95_ for gait speed (0.30 m/s) and step length (14.1 cm) in children were larger than those reported for the Gait Analyzer app in children and adolescents (gait speed = 0.14–0.15 m/s, step length = 8.3–9.5 cm)^30^. Lower MDC_95_ for gait speed in healthy adults (0.02–0.15 m/s) and patients with neurological diseases (0.13–0.14 m/s) have also been documented for smartphone apps focusing on standardized gait capacity measurements^39,42^. The MDC_95_ for gait speed measured with the Health app ranged from 0.20 to 0.30 m/s, which is above the minimum clinically significant difference (MCID) for usual gait speed estimated at 0.05 m/s in standardized walking tests^76,77^. This suggests that the absolute error in gait speed measurements is greater than this MCID, limiting the interpretability of subtle and meaningful changes in gait speed using the Health app and its suitability for clinical use.

The study has some limitations. First, the sample size for each age group was small, especially for the analysis of test–retest reliability in children due to non-expected missing data. Second, the multi-IMU-based APDM Mobility Lab was used as an external reference standard, which is not considered a gold standard for gait analysis. However, it has been demonstrated good to excellent concurrent validity against video motion capture systems and instrumented walkways^13–15^. Third, spatio-temporal gait parameters were captured during a 6MWT. Future studies needs to assess the validity and reliability of the Health app for measuring these parameters in more unprescribed and unsupervised context during longer observational periods of time to be considered representative of daily-life gait performance. Fourth, the wearing position of the iPhone was standardized for all participants (right pants pocket), but not the pants to be worn, which may have resulted in different signal quality for data processing across participants and repeated measurements. Fifth, the partially different test environments (indoors vs. outdoors) across the age groups hamper direct between-group comparisons of the validity and reliability results. Sixth, as the Health app does not provide access to individual gait cycles, the comparison with the APDM Mobility Lab was based on the mean values of gait parameters determined fully automatically by both systems, without being able to perform a precise manual time alignment of individual gait cycles between both systems prior to automatic data analysis. Lastly, findings are limited to healthy, fit and physically active persons. Future studies are needed to validate the gait measurements of the Health app in other populations with disability and/or lower gait performance.

In conclusion, the Heath app on iPhone has been shown to be valid and reliable for measuring gait speed and step length in adults and seniors. Children's gait speed can also be measured validly, but less reliably than in the adult age groups. Limited validity and reliability were documented for the measurement of step length in children. Even though the measurements of double support time were reliable in all age groups, they should be viewed with caution having consistently shown only low to moderate validity. Further, relatively large changes in the gait speed measurements of the Health app seem to be required to be confident that a real change has occurred. Reducing artifactual smartphone movements by tightly coupling the iPhone near the center of mass appears to be critical to reliably receiving gait parameter estimates from the Health app. Overall, the findings of this study suggest that the freely accessible and simple-to-use Health app on an iPhone carried in tight front pants pocket might be a valid and reliable tool for fully automated, unobtrusive, and continuous daily-life monitoring of gait speed and step length in adults and seniors.

## Data Availability

The datasets used and analyzed during the current study are available from the corresponding author on reasonable request.
